# The Perfect-Circle Technique Demonstrates Poor Inter-Rater Reliability in Measuring Posterior Glenoid Bone Loss on Magnetic Resonance Imaging

**DOI:** 10.1016/j.asmr.2024.100889

**Published:** 2024-02-05

**Authors:** Nata Parnes, Kyle J. Klahs, Alexis B. Sandler, Emily I. Wynkoop, Adam Goldman, Keith Fishbeck, Robert H. Rolf, John P. Scanaliato

**Affiliations:** a Department of Orthopaedic Surgery and Rehabilitation, Carthage Area Hospital, Carthage, New York, U.S.A.; b Department of Orthopaedic Surgery and Rehabilitation, Claxton Hepburn Medical Center, Ogdensburg, New York, U.S.A.; c Department of Orthopaedic Surgery, Texas Tech University Health Science Center, El Paso, Texas, U.S.A.; d Beacon Orthopaedics & Sports Medicine, Cincinnati, Ohio, U.S.A.; e Midwest Orthopaedics at Rush University Medical Center, Chicago, Illinois, U.S.A.

## Abstract

**Purpose:**

To evaluate the reliability of the “perfect-circle” methodology for measurement of glenoid bone loss with magnetic resonance imaging (MRI) in patients with posterior glenohumeral instability.

**Methods:**

A prospective chart review was performed on patients who underwent isolated arthroscopic posterior labral repairs in our institution’s electronic medical records between January 1, 2021, and June 30, 2021. Inclusion criteria included isolated posterior shoulder instability with posterior labral repair and corroborated tears on MRI. A total of 9 raters, either sports or shoulder and elbow fellowship-trained orthopaedic surgeons, each evaluated the affected shoulder MRI scans twice, at over 2 weeks apart. Measurements followed the “perfect-circle” technique and included projected anterior-to-posterior (AP) glenoid diameter, amount of posterior bone loss, and percentage of posterior bone loss.

**Results:**

Ten consecutive patients between the ages of 17 and 46 years with diagnosed posterior glenohumeral instability were selected. The average age was 28 ± 10 years, and 60% of patients were male. The patient’s dominant arm was affected in 40%, and 50% of cases involved the right shoulder. The average glenoid diameter was 29.62 ± 3.69 mm, and the average measured bone loss was 2.8 ± 1.74 mm. The average percent posterior glenoid bone loss was 9.41 ± 5.78%. The inter-rater reliability was poor for the AP diameter and for the posterior glenoid bone loss with intraclass correlation coefficients at 0.30 (0.12-0.62) and 0.22 (0.07-0.54) respectively. The intrarater reliability was poor for AP diameter and moderate for posterior glenoid bone loss, with intraclass correlation coefficients at 0.41 (0.22-0.57) and 0.50 (0.33-0.64), respectively.

**Conclusions:**

Using the “perfect-circle” technique for evaluating posterior glenohumeral bone loss has poor-to-moderate inter- and intrarater reliability from MRI.

**Level of Evidence:**

Level IV, prospective diagnostic study.

Posterior glenohumeral instability is an increasingly recognized cause of shoulder pain and dysfunction, particularly among young, athletic patients.^1^, ^2^, ^3^, ^4^, ^5^, ^6^, ^7^ While historically thought to comprise only 2% to 10% of cases of shoulder instability, recent studies have suggested that posterior instability may account for up to 24% of cases in certain active populations.^3^^,^^6^^,^^7^ Glenoid bone loss (GBL) in the setting of anterior glenohumeral instability has been well described; however, fewer studies have characterized GBL in patients with isolated posterior instability. Studies by Hines et al.^8^ and Wolfe et al.^9^ noted posterior GBL in 69% and 86%, respectively, of patients with primary, isolated posterior instability.

Biomechanical studies have shown that posterior GBL is correlated with greater posterior humeral translation in cadaveric models.^10^ Furthermore, Arner et al.^11^ found that greater or equal to 11% GBL implicated a 10 times’ greater surgical failure rate and greater or equal to 15% GBL was associated with a 25 times’ greater likelihood of failing following arthroscopic posterior labral repair.

Anterior GBL is commonly assessed using a best-fit “perfect-circle” method, first described by Sugaya et al.^12^ The “perfect circle” method relies both on the correct selection of the most representative sagittal slice as well as the measurements. While 3-dimensionally reconstructed computed tomography (3DCT) with humeral head subtraction is currently accepted as the gold standard for assessing glenoid morphology, these studies are costly to the patient and result in significant radiation exposure.^13^, ^14^, ^15^, ^16^, ^17^

In a study comparing the contour of the anterior and posterior glenoid using the “perfect-circle” technique on 3DCT reconstructions for estimating glenoid bone loss, Lansdown et al.^18^ concluded that estimation of anterior GBL based on the normal posterior glenoid rim may overestimate GBL because of differences in the contour of the anterior and posterior glenoid, whereas estimations of posterior GBL based on the anterior rim did not differ significantly from the intact glenoid. Several studies have demonstrated comparable measurements using magnetic resonance imaging (MRI) to 3DCT, and many surgeons now routinely use MRI in their practice to assess anterior GBL during preoperative planning.^19^^,^^20^ Although there is evidence that a best-fit “perfect-circle” method using MRI is effective for measurement of GBL in patient with anterior shoulder instability, there are no corresponding confirmatory data in a posterior instability scenario.^8^

The purpose of this study is to evaluate the reliability of the “perfect-circle” methodology for measurement of glenoid bone loss with MRI in patients with posterior glenohumeral instability. We hypothesized that our multisurgeon study would demonstrate good inter- and intrarater reliability, supporting the use of the “perfect-circle” technique for evaluating bone loss on preoperative MRI.

## Methods

This prospectively analyzed case series was designed as a multisurgeon validation study of the “perfect-circle” diagnostic methodology for estimating posterior glenoid bone loss.

### Patient Selection

Patients who underwent isolated arthroscopic posterior labral repair at a single high-volume shoulder/elbow and sports practice between January 1, 2021, and June 30, 2021 were identified. Inclusion criteria included a history of an isolated posterior glenohumeral instability, age younger than 50 years, and availability of a 3-Tesla MRI of the affected shoulder. Exclusion criteria included history of previous shoulder surgery or instability secondary to generalized hyperlaxity or posterior glenoid dysplasia (Walch B or C).

Ten consecutive patients who met the inclusion criteria were selected for this analysis. A surgeon (N.P.) who did not participate in the validation experiment served as a local study lead and ensured that the patient identifiers remained blinded to the assessors.

### Technique

All noncontrast MRIs of the shoulder were performed with the same imaging protocol on a 3-Tesla MRI system (sequences included: T1-weighted, T2-weighted, T2 fat-suppressed) and acquired in the plane of the glenoid. Images were assessed by 9 attending orthopaedic surgeons using Joints 5.37.0 PACS software (Medstrat, Downers Grove, IL). All surgeons were shoulder/elbow or sports fellowship-trained. All participating surgeons routinely manage posterior glenohumeral instability in practice, with approximately 216 procedures for posterior instability performed annually by the practice during the study period. All surgeons were familiar with, and routinely use, their picture archiving and communication system.

**Fig 1:**
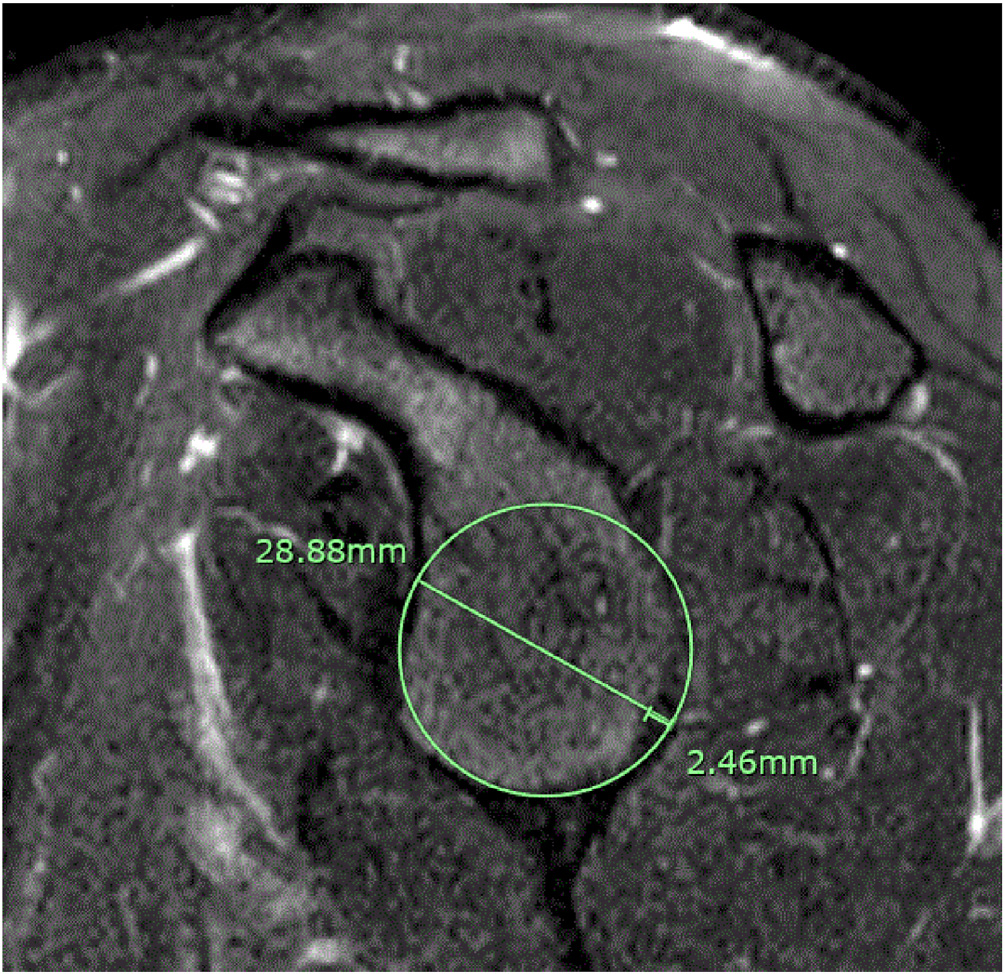
MRI sagittal view at the glenoid face of the left shoulder in the plane of the glenoid. (MRI, magnetic resonance imaging.)

All surgeons were provided a technique guide outlining the steps for estimating GBL using the “perfect-circle” technique, as described by Sugaya et al.^12^ The measuring surgeons were not told which series and image to obtain their measurements from and the best en-face sagittal MRI scan of the glenoid, in the opinion of the measuring surgeon, was used. To obtain measurements, a line was first drawn along the long axis of the glenoid. A best-fit circle based off the inferior two-thirds of the glenoid was then approximated (Fig 1). Total bone loss and percentage bone loss were calculated using the following procedure: to calculate total bone loss, a line was drawn perpendicular to the long-axis line spanning the perceived bone loss. The expected diameter was then calculated by drawing a line perpendicular to the long-axis line to create a measurement representing the diameter of the “perfect circle.” Percentage bone loss was calculated by dividing the measured diameter by the expected diameter and then multiplying by 100.

### Statistical Analysis

All statistical analysis were performed with RStudio 2023 (Posit Software, PBC, Boston, MA). Inter-rater reliability was determined by calculating intraclass correlation coefficients (ICCs) based on averaged measurements from investigators. Intrarater reliability was also determined from separate sets of measurements from each investigator performed over 2 weeks apart. ICC values were evaluated based on the thresholds proposed by Koo and Li^21^: a value less than 0.5 represented poor reliability, 0.5 to 0.75 represented moderate reliability, 0.76 to 0.9 represented good reliability, and 0.9 or greater represented excellent reliability.

## Results

Within the study period, 10 patients were selected for analysis. The average age was 28 ± 10 years, and 60% of patients were male. The patient’s dominant arm was affected in 40%, and 50% of cases involved the right shoulder (Table 1). The average glenoid diameter was 29.62 ± 3.69 mm, and the average measured bone loss was 2.8 ± 1.74 mm. The average percent posterior GBL was 9.41 ± 5.78% (Table 2). The inter-rater reliability was poor for the AP diameter and for the posterior GBL with ICCs at 0.30 (0.12-0.62) and 0.22 (0.07-0.54) respectively. The intrarater reliability was poor for AP diameter and moderate for posterior GBL with ICC at 0.41 (0.22-0.57) and 0.50 (0.33-0.64) respectively (Table 3).

**Table 1:** Study Population Characteristics

Average age, y, mean ± SD, range	28 ± 10, 17-46
% Male	60%
% Dominant arm	40%
% Right shoulder	50%

Patient	Age, y	Sex	Arm Dominance	Affected Side
1	19	F	Dominant	Right
2	43	F	Nondominant	Left
3	46	F	Dominant	Right
4	33	M	Nondominant	Left
5	17	M	Dominant	Right
6	27	M	Dominant	Right
7	36	M	Nondominant	Right
8	17	M	Nondominant	Left
9	19	M	Nondominant	Left
10	21	F	Nondominant	Left

F, female; M, male; SD, standard deviation.

**Table 2:** Average Glenoid and Bone Loss Measurements by Patient

Patient	Average Glenoid Diameter Using Perfect-Circle Technique, mm, range	Average Bone Loss, mm, range	Average Bone Loss, %, range
1	29.14 ± 4.3, 25.8-45.6	2.23 ± 1.8, 0-6.5	7.68 ± 6.2, 0-21.7
2	27.83 ± 2.35, 24-32.8	2.65 ± 1.35, 0.8-6.7	9.57 ± 5.07, 3.1-24.5
3	27.79 ± 5.07, 22.3-47.1	3.49 ± 0.63, 2.4-4.8	12.66 ± 2, 9-14.6
4	31.23 ± 3.02, 26.4-36	4.17 ± 2.01, 0.7-7.3	13.55 ± 6.95, 2.4-27.7
5	28.9 ± 2.05, 24.6-33	3.11 ± 1.33, 0-6.4	10.67 ± 4.41, 0-20.8
6	29.48 ± 2.28, 26-34.8	2 ± 1.41, 0-4.1	6.69 ± 4.73, 0-13.4
7	28.97 ± 4.39, 22.3-44.7	1.85 ± 1.26, 0-4.7	6.39 ± 4.53, 0-17
8	33.76 ± 1.08, 31.9-35.8	3.79 ± 1.59, 0-7	11.21 ± 4.63, 0-20.3
9	30.97 ± 3.48, 20-35.8	2.85 ± 6.31, 0-7.3	8.93 ± 6.31, 0-20.9
10	28.17 ± 2.09, 22.5-30.7	1.88 ± 1.52, 0-6.4	6.78 ± 5.88, 0-25.6
Total average	29.62 ± 3.69, 20-47.1	2.8 ± 1.74, 0-7.3	9.41 ± 5.78, 0-27.7

**Table 3:** Inter- and Intrarater Reliability

	ICC	95% CI
Inter-rater reliability		
Perfect-circle diameter	0.299	0.115-0.624
Posterior bone loss	0.222	0.069-0.543
Intrarater reliability		
Perfect-circle diameter	0.41	0.223-0.569
Posterior bone loss	0.50	0.330-0.640

CI, confidence interval; ICC, intraclass correlation coefficient.

## Discussion

Within this study, a single observer demonstrated only poor to moderately reliable and between different observers there was poor reliability when assessing posterior GBL using the “perfect-circle” technique on MRI. These findings do not support our hypothesis and suggest that the “perfect-circle” methodology may represent a suboptimal technique for assessing posterior GBL with MRI.

GBL is a well-described consequence in the majority of patients with posterior shoulder instability.^1^^,^^8^ A study by Bedrin et al.^22^ demonstrated that a single posterior instability event results in a mean 5.4% GBL. The amount of GBL after a recurrent posterior glenohumeral instability event was greater than that after first-time instability. Properly identifying and characterizing GBL is critical step in the preoperative planning process for patients with posterior instability, as GBL is a known risk factor for the failure following soft-tissue stabilization.^23^^,^^24^ It therefore follows that the validity of the “perfect-circle” technique is an important aspect of one’s treatment algorithm, as correct quantification of bone loss allows for selection of the most appropriate surgical procedure. However, our findings suggest that despite following a standardized protocol for obtaining measurements, the inter-rater reliability among 9 fellowship-trained orthopaedic surgeons for the AP diameter and GBL is poor. This is of particular concern, given the increasing evidence that even subcritical bone loss to the low teens may predispose patients to poor outcomes following isolated posterior Bankart repair.^11^ Failure to recognize critical or subcritical GBL may lead surgeons to perform soft-tissue stabilization in cases in which bone block reconstruction may be more appropriate.^11^^,^^25^

Our findings are of particular interest in the context of existing literature. A study by Weber et al.^26^ demonstrated that the measuring GBL differed significantly among reviewers and represented a source of error when assessing GBL in patients with anterior shoulder instability on both MRI (ICC, 0.8) and CT (ICC, 0.91). While several other studies have reported more favorable ICCs using this technique, their methods preclude the generalization of their findings to routine clinical practice.^19^^,^^20^ Sgroi et al.^20^ reported good-to-excellent reliability assessing the ability of MRI to characterize bony Bankart lesions. However, their study was limited to 2 resident physician observers, and it is unclear as to whether readers were able to select their own sequences and images for measurement. Chalmers et al.^27^ demonstrated good inter-rater reliability for GBL measurements from MRI; however, this was derived from measurements by 2 surgeons who were given preselected MRI slices from which to measure, which only investigates half of the “perfect-circle” technique. Furthermore, the authors noted that despite ICCs within an acceptable range, differences reported between the 2 observers would still have resulted in different treatment recommendations in more than 30% of cases in their analysis. Simply put, measurements affecting surgical planning should not vary by observer in such a high proportion of cases. These findings call attention to the need for more reliable methods of assessing GBL and the authors echo Chalmers et al. in calling for further research into alternative methodology for assessing glenoid bone loss. Also, Moroder et al.^28^ identified that scapula tilt contributes to the impreciseness when measuring GBL.

Another possible reason for the poor reliability of the “perfect-circle” technique to assess GBL in patients with posterior as compared with anterior shoulder instability is the asymmetric differences in glenoid injury morphology. Livesey et al.^29^ compared GBL patterns in a matched cohort of patients with anterior versus posterior glenohumeral instability. In their study, they found that posterior GBL occurred more inferiorly and at an increased obliquity compared with anterior GBL. This pattern was consistent for traumatic and atraumatic posterior GBL. In particular, the morphology of posteriorly angulated and sloped bone defects is distinct from lesions typically seen in anterior instability, where bone is fractured or compressively lost and can readily be measured as a distinct near 90° transition out of plane on advanced imaging. In contrast, posteriorly sloped bone loss occurs from erosive, repetitive, posterior loading and may not appear as distinct bone loss lesions when calculating the total area of loss on sagittal imaging.^30^

Lastly, Moroder et al.^28^ assessed the effect of scapula tilt and best-fit circle placement when measuring GBL in patients with shoulder instability. They concluded that impreciseness of scapula positioning for creation of an en face view of the glenoid as well as varying best-fit circle placement significantly altered glenoid defect size measurement results. In patients with a dysplastic or hypoplastic glenoid and patients with severe glenoid retroversion, precise scapular positioning can be challenging.

The poor inter-rater reliability demonstrated by this analysis is relevant within contemporary literature. Many existing studies reporting risk factors for GBL in posterior shoulder instability as well as operative techniques for the management of instability rely on the “perfect-circle” technique for patient selection. However, if surgeons do not reliably measure the same degree of GBL, the ability to generalize these findings becomes limited.

### Limitations

There were several limitations to this study. A small sample size lessens the result power as well potentially introduces bias. We also could not evaluate the accuracy of measurements for GBL, as we did not have gold standard 3DCT imaging to compare. We did not account for inconsistencies in the slice the rater selected to perform the measurements, which would have drastic effects on reliability. Because of the multiple steps between MRI and measurements, it is impossible to definitively pin the poor inter- and intrarater reliability solely on the “perfect-circle” technique.

## Conclusions

Using the “perfect-circle” technique for evaluating posterior glenohumeral bone loss has poor-to-moderate inter- and intra-rater reliability from MRI.

## Disclosure

The authors (N.P., K.J.K., A.B.S., E.I.W., A.G., K.F., R.H.R., J.P.S.) report no conflicts of interest in the authorship and publication of this article. Full ICMJE author disclosure forms are available for this article online, as supplementary material.

## Supplementary Data

ICMJE author disclosure forms
